# Impact of Adverse Drug Reactions in Patients with End Stage Renal Disease in Greece

**DOI:** 10.3390/ijerph17239101

**Published:** 2020-12-06

**Authors:** Marios Spanakis, Marianna Roubedaki, Ioannis Tzanakis, Michail Zografakis-Sfakianakis, Evridiki Patelarou, Athina Patelarou

**Affiliations:** 1 Department of Nursing, Faculty of Health Sciences, Hellenic Mediterranean University, Estavromenos, GR-71140 Heraklion, Crete, Greece; mspanakis@hmu.gr (M.S.); mzografakis@hmu.gr (M.Z.-S.); epatelarou@hmu.gr (E.P.); 2 Computational BioMedicine Laboratory, Institute of Computer Science, Foundation for Research and Technology—Hellas (FORTH), GR-70013 Heraklion, Crete, Greece; 3 Department of Nephrology, General Hospital of Chania, Meletiou Metaxaki 25, GR-73131 Chania, Crete, Greece; mariannarubedaki@yahoo.com (M.R.); ioatzan@gmail.com (I.T.)

**Keywords:** chronic kidney disease, end-stage renal disease, adverse drug reactions, drug–drug interactions, quality of life, chronic disease

## Abstract

Background: Patients with end-stage renal disease (ESRD) require specialized therapeutic interventions. The decreased renal function that modulates the physiology and presence of comorbidities is often associated with variations in the pharmacological response, thus increasing the risk of adverse drug events or reactions (ADE/ADRs) from co-administered drugs. Methods: A cross-sectional study to record comorbidities, drug–drug interactions (DDIs), ADE/ADRs in patients with chronic kidney disease of stage five in Greece. The study enrolled 60 patients of mean age 64.8 ± 12.9 years, undergoing hemodialysis three times a week. Demographic and social factors, comorbidities, laboratory test data, medication regimens, DDIs and the reporting of ADE/ADRs were analyzed. Results: Cardiovascular diseases and diabetes were the main comorbidities. In total, 50 different DDIs of various clinical significance were identified. CNS, GI-track, and musculoskeletal-system-related ADE/ADRs were most often reported by patients. ADE/ADRs as clinical outcome from DDIs were associated in 64% of the total identified DDIs. There was a positive trend between number of medications, ADE/ADRs report and DDIs. Conclusions: The impact of ADE/ADRs in ESRD patients should be always considered. Guidelines as well as continuous training in the context of evidence-based clinical practice by healthcare personnel on therapy administration and prevention of adverse events are important.

## 1. Introduction

Chronic kidney disease (CKD) is characterized from a progressing reduction in renal function, and end-stage renal disease (ESRD) patients end up requiring renal replacement therapy (RRT). CKD is an important health issue worldwide with high morbidity and mortality rates among the non-communicable diseases. The all-cause mortality due to CKD or to CKD-attributable cardiovascular diseases is estimated to account for 5% globally, and its prevalence is growing [1,2,3]. Considering ESRD patients, demographics data from several studies reveal a tendency to increase the size of the patient population as well as their age, highlighting the importance of advanced nephrological healthcare [4]. Regarding Greece, epidemiological data show a high incidence and prevalence rate for ESRD patients, with approximately 0.5–1 million patients and 1500 deaths related to CKD [2,5].

CKD patients embody a clinical group that requires special therapeutic interventions to treat the complications and co-existing comorbidities such as diabetes, cardiovascular diseases, metabolic bone syndrome and anemia. The reduction in renal function leads in changes in the proper function of other organs that subsequently impacts pharmacokinetic (PK) and pharmacodynamic (PD) parameters and processes of administered medications [6]. Due to reduced glomerular filtration rate (GFR), the renal clearance of drugs is diminished while changes in renal function also affect metabolic clearance from the liver. ESRD patients that undergo hemodialysis have changes in drug absorption processes attributed to increased paracellular leakage and decreased efflux transporter activity in the gastrointestinal track (GI-track); drug accumulation and higher free drug concentrations due to decreased albumin levels and competition for protein binding by uremic toxins; reduced intrinsic clearance from liver cytochromes P450 (CYPs); decreased biliary excretion; lower hepatic blood flow and changes in tissue composition (i.e., water/lipid partition) [7,8,9]. All these factors contribute to changes in pharmacological response and require specific strategies for drug administration and therapeutic regimens in order to minimize any occurrence of negative events from the use of medical products or adverse drug events (ADEs) and, more importantly, noxious and causally related drug responses or adverse drug reactions (ADRs), while increasing safety and efficacy [10,11].

ADE/ADRs are an important issue for healthcare provision, since they can be the cause of hospital admissions and re-admissions, thus prolonging hospitalization as well as in-hospital morbidity and mortality. In addition, the presence of ADRs adversely affects patients’ quality of life which also impacting patients’ adherence and compliance [12,13,14]. Patients with CKD, especially ESRD, are particularly exposed to potential ADE/ADRs because they usually receive a variety of medications and have multiple comorbidities [15,16,17]. In addition, ADEs/ADRs could be related to their disease state and complications from the disease progression or from results of the therapeutic procedures of hemodialysis. The complexity of medication regiments and the high number of drugs that are administered in ESRD patients can also lead to the appearance of drug–drug interactions (DDIs) that can be the pharmacological underlying reason for ADRs [18,19]. Overall, the presence of ADRs in ESRD patients can be related to impaired quality of life, possible hospital re-admissions in case of life-threatening ADRs, poor adherence and compliance and increased healthcare costs [14,20,21,22,23,24].

In this context, the European Union (EU) has launched several activities regarding pharmacovigilance and drug-safety monitoring in effort to improve the healthcare eco-system and analyze data from different member states [25]. However, for Greece, data are scarce, particularly for special patient population groups such as CKD patients. Hence, the aim of this work focused on ESRD patients in Greece in an effort to record comorbidities, analyze the administered medical regiments for clinically significant DDIs and record ADE/ADRs as they are described in national formulary and summaries of product characteristics (SOPs) of drugs.

## 2. Materials and Methods

### 2.1. ESRD Patients and Study Information

The cross-sectional study was conducted at the department of Nephrology of the General Hospital of Chania (GSC), Crete, Greece. The study was conducted in accordance with guidelines for reporting observational studies (Strengthening the Reporting of Observational Studies in Epidemiology (STROBE)) and Evidence-Based Practice approaches [26]. The study was approved by the hospital’s ethics committee (No. 11/29-08-18) and the 7th Health Region of Crete Directorate of Public Health (No. 18022). All the study’s procedures were carried out following the rules of the Declaration of Helsinki of 1975 and were in accordance with the general data protection regulation (GDPR) [27]. The participants in the study were given an information brochure and included in the study after they singed the informed consent form. The data collection was conducted through structured questionnaires. The STROBE information is briefly given in Table 1.

The study was conducted in, subsequently, two out of the three different sessions during the weekly visit of ESRD patients in the clinic. Patients that signed the informed consent to participate in the study were initially interviewed to gather their demographic data and gave access to their medical records for laboratory test results and medication regiments. In the second session, patients were asked to choose from the list of ADE/ADRs provided to them, the ones that they experience and then were briefly interviewed regarding which drug(s) can be related with the reported ADE/ADRs based on the Naranjo Scale to assist in evaluation of any causality for the reported ADE/ADRs [28].

### 2.2. Drug–Drug Interactions

Potential drug–drug interactions were detected using Medscape [29] and drugs.com [30] as primary sources. The clinical significance of the identified interactions was based on the availability of scientific data from the literature that provide sufficient evidence of the underlying biological mechanism and information regarding the handling of the co-administration. The clinical significance of the interactions is represented as a ‘Serious-use alternative’ for those where specific actions should be applied, ‘monitor closely’ for those where precautions should be taken in some cases, and ‘moderate-minor’ for minor ones.

### 2.3. Adverse Drug Events (ADEs) and Adverse Drug Reactions (ADRs)

ADE/ADRs were extracted from the Greek National Formulary of Drugs as well as from European Medicine Agency (EMA) and available summaries of products characteristics (SPC). Based on the information available, eight distinct classes based on organ systems were used to categorize the ADE/ADRs (Table 2). The ADE/ADRs that are included in the questionnaires are derived from the Formulary and SPCs, and it is considered that they are causally related with drug administration. However, due to disease complications and in an effort to further evaluate ADE/ADRs, the Naranjo Scale was employed and patients were asked to provide answers in order to assist in the evaluation of any causality for the reported ADE/ADRs.

## 3. Results

### 3.1. Demographics, Comorbidities, and Lab Test Results

A total number of 60 patients with ESDR signed the informed consent form and were included in the study. From the 60 ESRD patients, 68.3% (*n* = 41) were male and 31.7% (*n* = 19) female. The average age of ESRD patients were 64.8 ± 12.9 years old with 51.7% of them characterized as elders (above 65 years old). The average body mass index (BMI) was calculated at 30.6 ± 5.6 (kg/m^2^) and the dry weight at 76.0 ± 17.8 kg (Table 3). A negative characteristic was the relatively high prevalence of smoking (36.7%) among the participants.

**Figure 1:**
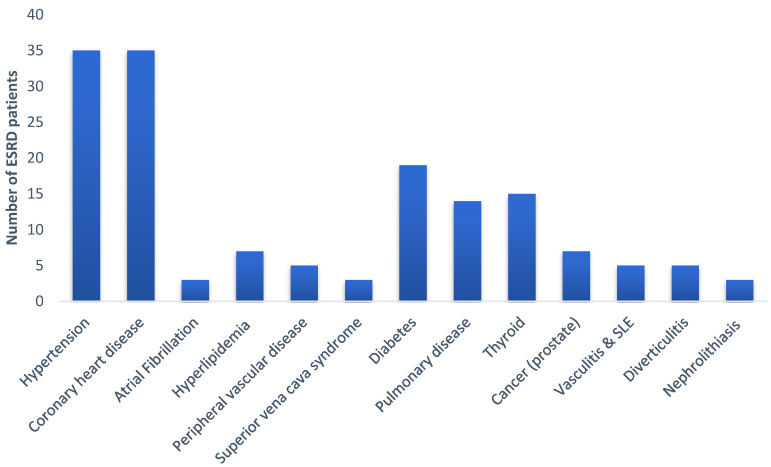
Comorbidities recorded among the ESRD patients of the study (ESRD: End Stage Renal Disease; SLE: Systemic Lupus Erythematous).

The average disease period for the studied population was 4 ± 3 years. The comorbidities present along with ESRD are illustrated in Figure 1. The most often comorbidity was found for hypertension (*n* = 35, 58%) and coronary heart disease (*n* = 35, 58%) whereas, as expected, diabetes (*n* = 19, 31.7%) and other cardiovascular diseases were also prevalent. Additionally, comorbidities such as pulmonary disease, hematologic diseases, inflammation diseases and cases of prostate cancer were also recorded among the ESRD patients (Figure 1).

### 3.2. Drug Categories and Drug–Drug Interactions

**Figure 2:**
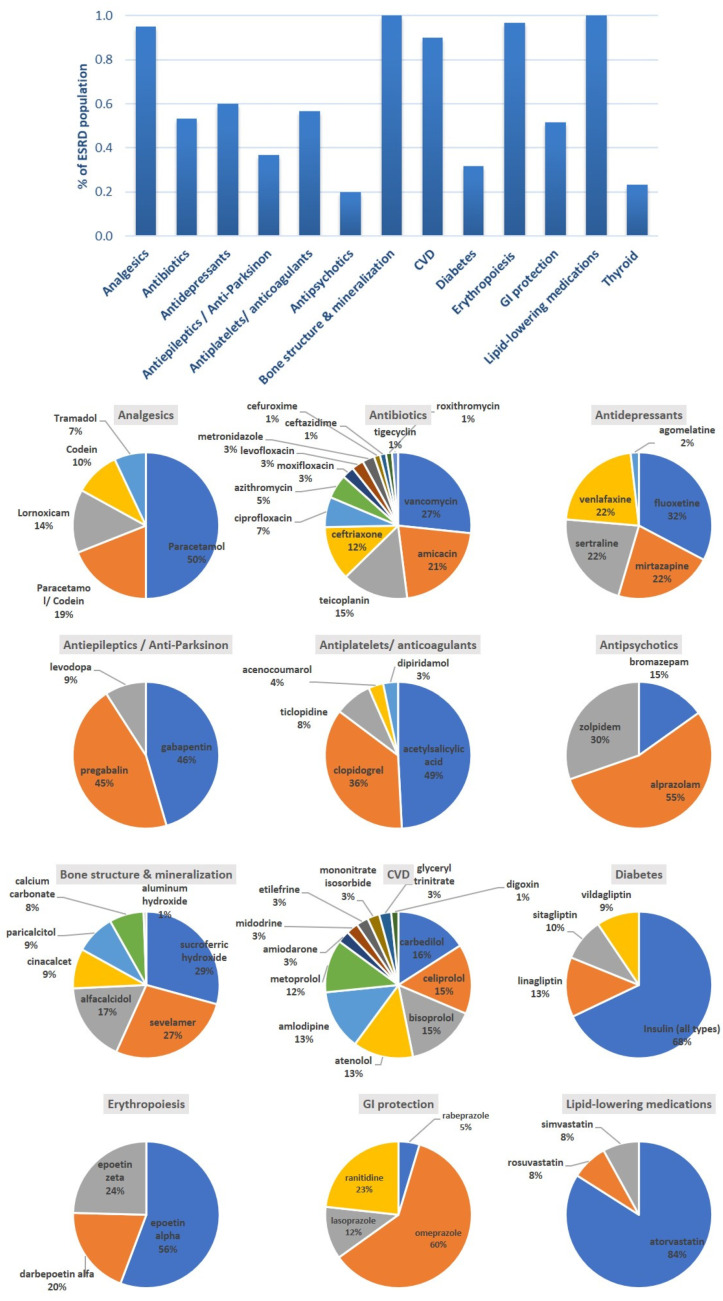
Distributions of medications administered among the ESRD patients. Bar graphs: drug categories among patients; chart pies: pharmacological compounds from each category (Thyroid drugs refer to levothyroxine (100%) alone and not included as pie) (ESRD: End-stage Renal Diseasel CVD: Cardio-Vascular Disease; GI: gastro-intestinal).

All ESRD patients were prescribed with phosphate-binding agents, drugs for bone structure and mineralization as well as lipid-lowering medications (*n* = 60, 100.0%). In addition, almost all of them were receiving therapies for anemia (*n* = 58, 97%) analgesics or nonsteroidal anti-inflammatory drugs (*n* = 57, 95%) for pain management related to the ESRD. Overall, patients received an average of 10 ± 3 different medications (minimum four and maximum 18 different drugs). The prevalence of hypertension (and generally CVDs) resulted in recording an increased number of patients (*n* = 54, 90%) receiving relative medications, as well as antiplatelet/anticoagulant therapies (*n* = 34, 57%). CVD drugs referred to antihypertensive agents (58%), mostly β-blockers (61.7%) and statins (60%). Patients were often prescribed with CNS drugs such as antidepressants (*n* = 36, 60%) and antipsychotics (*n* = 12, 20%). Other medications recorded among ESRD patients were related with type I or type II diabetes and thyroid medications (levothyroxine, *n* = 14, 23%), as well as antiepileptics and anti-Parkison medications (Figure 2, bar graphs’ top). All medications were administered following the relative clinical guidance for dose adjustment according to GFR and RRT data. Regarding water restriction, all patients followed a diet with approximately 250 mL water per day and 250–500 mL from diet (food, juices, coffee etc). This is the average recommendation adjusted based on personalized needs and characteristics such as dry weight, comorbidities, monthly laboratory test results, diet, and quality of life.

**Figure 3:**
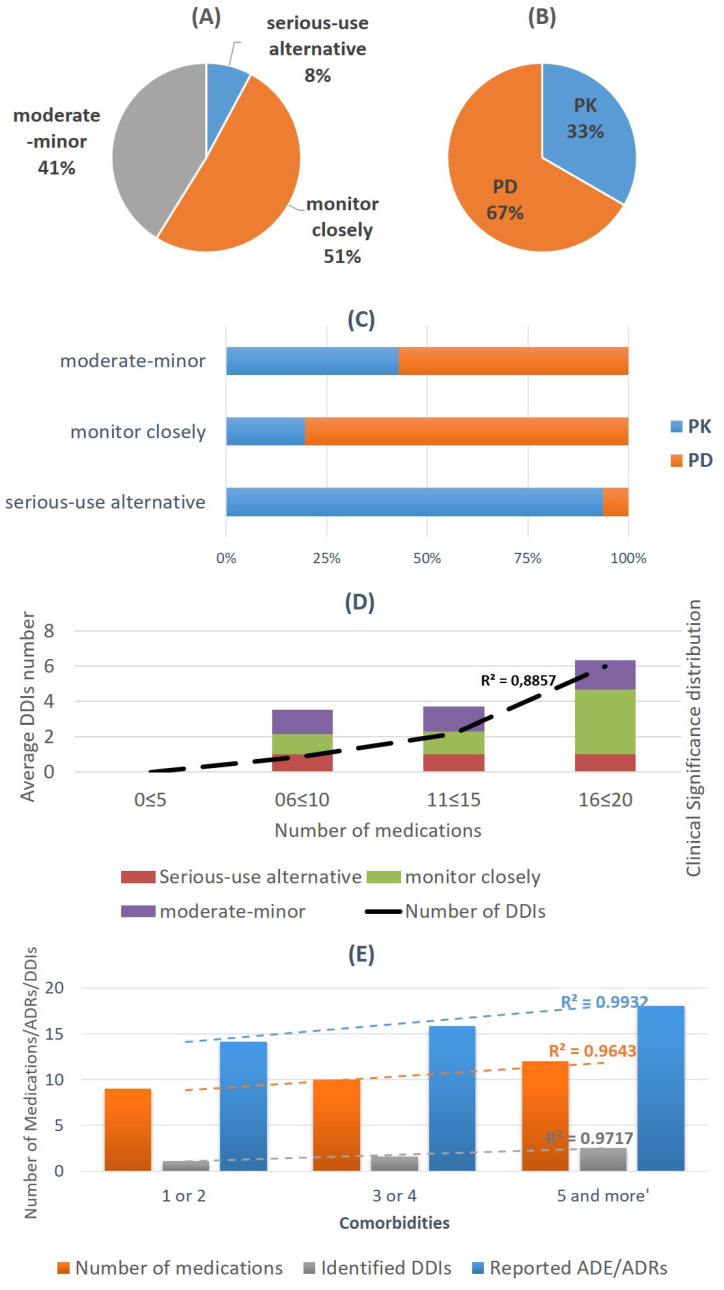
Identified DDIs and (**A**) Clinical significance; (**B**) pharmacological process involved; (**C**) Clinical significance and underlying pharmacological mechanism (**D**) Trends in number of DDIs over number of medications (dashed line) and the clinical significance distribution per average number of identified interactions (bars) (**E**) Impact of comorbidities and medication number on identified DDIs and ADE/ADRs occurrence (DDIs: Drug-Drug Interactions; ADE/ADRs: Adverse Drug Events/Adverse Drug Reactions).

DDIs were assessed through Medscape and drugs com. Although for some patients (*n* = 15, 25%) no interactions with clinical relevance were detected, for the rest 75% (*n* = 45), there was an average prevalence of 2 ± 1 DDIs for each patient (total average 1.6 ± 1 considering all ESRD patients). Regarding interacting medications, 50 unique drug combinations were detected that could be related with clinical significant DDIs, with 8% of them considered serious, 41% as needing close monitoring and the remaining 41% of moderate–minor clinical significance (Figure 3, Table 4). The underlying pharmacological mechanisms were referred to as PK interactions for 33% of the cases and the remaining 67% were PD-related. Regarding the impact of pharmacological process affected and the clinical significance, it was evident that PK interactions could lead in serious interactions, in which case alternatives or precautions should be considered, whereas PD related mechanisms were for cases in which monitoring should be applied for ESRD patients. PK-related interactive mechanisms were mostly referring to the modulation of gastrointestinal (GI) absorption that could alter drug bioavailability (F), resulting in reduced absorption, and thus reduced pharmacological action or inhibition of liver metabolism from cytochromes-P450 such as CYP3A4, CYP2C19 and CYP2D6 that could lead to enhanced PD action, and thus ADRs, or reduced PD action in the case of active metabolites. PD-relative mechanisms were mostly synergy mechanisms that could enhance the PD action for the co-administered drugs, and thus lead to clinically significant ADRs (Table 4).

There was a positive trend (r^2^ = 0.882) regarding the average number of DDIs detected and number of medications administered, revealing that as the number of medications rises, the risk for DDIs also increases. In addition, the clinical significance for monitoring the outcome of potential DDIs also showed increasing trends when the total number of medications exceeded number 15 (Figure 3). In addition, patients can be stratified according to the number of comorbidities in three groups, with a positive trend in the number of medications, DDIs detected and ADE/ADRs reported.

### 3.3. ADE/ADRs Recorded

**Figure 4:**
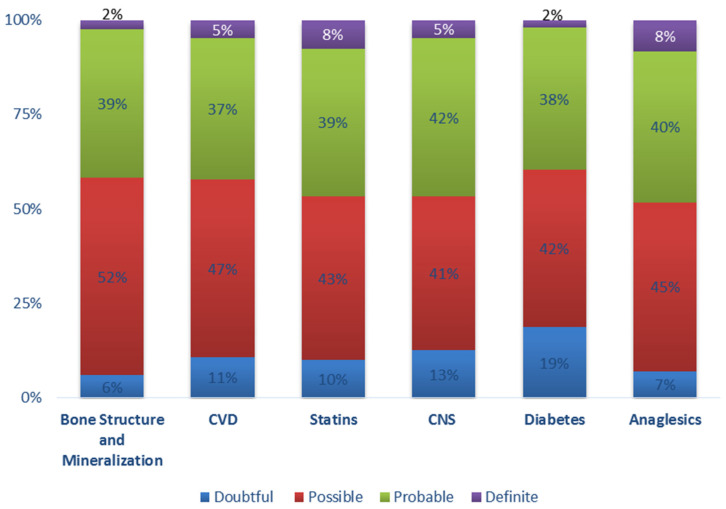
Naranjo scale results for different drug categories prescribed in ESRD patients of the study (CNS: central nervous system drugs including antiepileptics, antipsychotics, antidepressants, anti-parkinson; CVD: cardiovascular disease drugs, including antiplatelet–anticoagulant).

**Figure 5:**
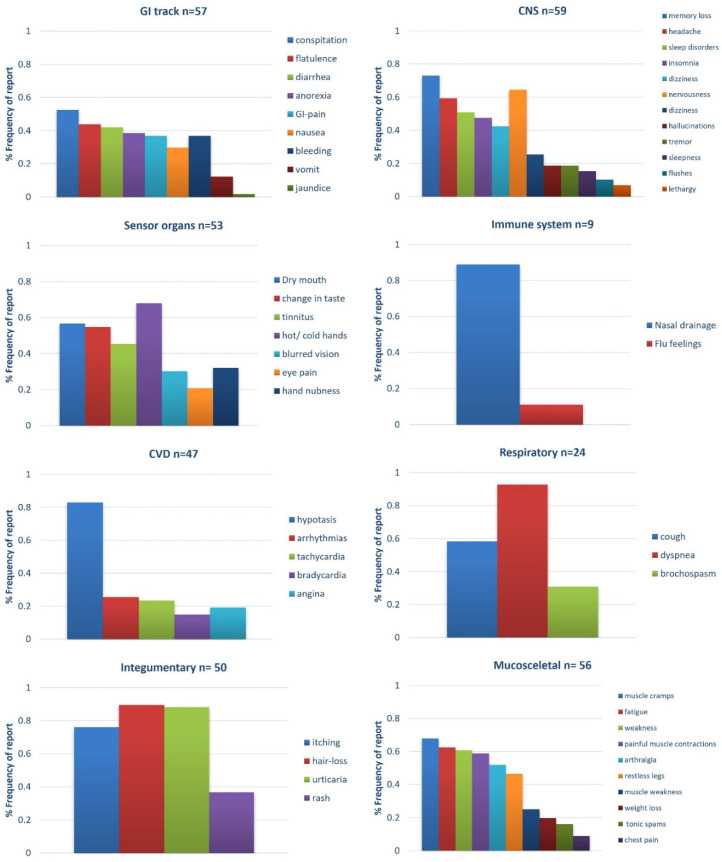
Distribution and frequency of reported ADE/ADRs in ESRD study group in relevance with related organ systems (GI: gastro-intestinal; CNS: Central Nervous System).

All 60 patients reported at least one or more ADE/ADRs from the list, provided with average 16 (min = 9, max = 26) ADE/ADRs per patient. Figure 4 depicts the Naranjo scale for the most frequent drug categories where ESRD patients relate administration of a drug with ADE/ADRs appearance, whereas Figure 5 illustrates the distribution of recorded ADE/ADRs regarding the related system as they were categorized in this study (see Table 1). The Naranjo ADE/ADRs probability scale suggests that most cases of the recorded ADE/ADRs were possible or probable in causation with drug administration, although they can convey patients’ report bias. The ADE/ADRs most often reported were related to CNS system such as memory loss (*n* = 43, 72.9%) and headaches (*n* = 35, 59.3%). In addition, a high prevalence of ADE/ADRs was reported regarding GI track and regarding constipation (*n* = 30, 52.6%) and swelling (*n* = 25, 43.9%). Musculoskeletal system ADE/ADRs reported those of muscle cramps (*n* = 38, 67.9%) and fatigue (*n* = 35, 62.5%). ADE/ADRs related to sensor organs (eyes, ears, etc.) were stated to be mouth dryness (*n* = 30, 56.6%) and change in taste (*n* = 29, 54.7%). ADE/ADRs related with integumentary system such as itching (*n* = 38, 76%) and cough (*n* = 14, 23.7%) were also evident. CVD-related ADE/ADRs were mainly regarding hypotension issues (*n* = 39, 66.1%). ADE/ADRs for respiratory or immune system were described to a lesser extent.

## 4. Discussion

The aim of this cross-sectional study was to record the parameters related to the occurrence of ADE/ADRs from the medication regiments in patients with ERSD in Greece following evidence-based approaches. Apart of the physiology changes from disease progression, it appears that patients on dialysis have a high level of medication burden, which can be related to the frequent occurrence of side effects associated not only with disease complications but also with administered medication or therapy procedures such as hemodialysis.

The 60 ESRD patients that were included in the current study had similar characteristics in terms of demographic, dry weight, and lifestyle habits, with data available from previous studies [23,31,32,33]. The frequency of comorbidities such as CVD and diabetes (type I and II) were also evident and, in the current study, ESRD patients showed a high prevalence of CVD-related diseases (Figure 1). CKD is often accompanied with 2–3 additional comorbidities such as hypertension (or other CVD) and diabetes [34]. These comorbidities have been causally related to the occurrence of CKD in the elder population (>65 years old). Generally, cardio-nephrosis syndrome, especially in patients with CKD, has a 10–20 times higher risk of mortality compared to the general population without renal disease [35].

There was an increased record of prescribed medication among ESRD patients with approximately 10 ± 3 medications per patient, a number which is comparable with other studies [31]. The analysis of the medication regimens showed that the increased number of medication and the presence of additional comorbidities could be related to the appearance of clinically significant DDIs, thus increasing the likelihood of developing side effects from administered therapy, which was also evident from the increased frequency of ADE/ADRs report, as well as the risk of reduced patient compliance with the resulting increased likelihood of re-hospitalization [24,36,37].

ESRD patients are susceptible to DDIs due to physiology changes which, apart from kidney function, change the function of other organs such as liver and GI-track (i.e., metabolizing enzymes and transporters) [8]. In this study, a positive correlation was observable between the increased number of medications, the number of ADE/ADRs reported and the DDIs found [36,37]. In the current study, one frequent interaction with increased clinical significance was the co-administration of sucroferric oxyhydroxide (SOH) with levothyroxine [38]. SOH is an iron-based phosphate binder (PB) used for the control phosphate levels in serum for adult CKD patients. According to the product’s SPC and the literature data, the interacting mechanism involves the chelation of oral thyroid hormones resulting in an insoluble complex that is poorly absorbed within the GI tract, and thus levothyroxine should be administered with a more than 4 h gap prior to the use of SOH. Generally, among the recorded DDIs, it was evident most of them could be related to the reported ADE/ADRs due to the modulation of either PK or PD processes. The presence of ADE/ADRs from the musculoskeletal system, for example, reported in patients receiving statins, could be related to the co-administration of nifedipine or azithromycin in that cases (Table 4). Muscle cramps, muscle weakness and myalgia were found to be more often reported in patients treated with statins, where it could be attributed to the possible interaction with co-administered drugs in combination with progressed renal failure [39,40]. Another consideration should be made for potential interactions with CNS drugs such as alprazolam that can enhance the PD effects of the drug in those patients [41,42]. Generally, CNS and CVD medications (including statins and anti-platelet, anticoagulant drugs) can be the possible or probable reason for ADE/ADRs in the current population, as also estimated from the Naranjo scale results (Figure 5) [43,44,45].

The results of the study regarding ADE/ADRs showed that those related to CNS were common among the ESRD population with 59/60 (98.3%) showing from one to three side effects (memory loss, headache, and sleep disorders). Ischemic cerebrovascular disease and underlying microvascular pathology appear to play a key role in the pathophysiology of cognitive impairment in patients with CKD [46]. Dialysis seems to cause an increased occurrence in episodes of acute cerebral ischemia and especially during it, while it requires increased awareness regarding medication. The occurrence of headaches in 59.3% of the respondents, despite taking analgesic and anti-inflammatory drugs (of these, 83.3% received only paracetamol, 31.7% paracetamol and codeine and 16.7% only codeine). In the present study, the incidence of headache was recorded as a result of drug action and not as a result of ultrafiltration during dialysis without investigating the clinical features of headaches and their association with factors such as anxiety and depression. However, the rate of reported headache was quite high and it is a side effect feature that has been correlated with several activating factors, and several strategies have been proposed to reduce it [47]. Moderate to severe headaches are a common neurological symptom in this group of patients, ranging from 28–70% with hypotension and redistribution in urea homeostasis, which are factors under investigation. In the category of side effects from the nervous system, sleep disorders were also observed in 50.8% of patients with insomnia, where the patients reported that they had difficulty sleeping with frequent awakenings during the night. Medication was associated with the intensification of insomnia, headache, dry mouth, and especially restless legs syndrome [41,48].

The effects on the GI-track related symptoms appear to worsen patients’ already aggravated condition, mainly by taking phosphorus-binding pills, food, and water restriction, uremic profile and other medication, and are like other data categorizing ADE/ADRs such as diarrhea, constipation and abdominal discomfort and distention as mild and transient [49]. During the present study, patients reported generalized disorders that were thought to be due to medication such as fatigue, dry mouth, and changes in taste. It was quite difficult to distinguish between the reported disorders in terms of the clinical features of renal insufficiency and the secondary effects of multiple medication. The disease can affect a wide range of systems, resulting in nervous, CVD, GI-track and hematopoietic complications. Fatigue in patients undergoing dialysis is considered a normal side effect and is often attributed to diseases such as anemia, inflammation, medication and the intensity and frequency of dialysis [50]. In addition, a main finding in the category of skin disorders was the itching that appeared in 76% of the respondents. Uremic pruritus may be focal or generalized, affecting patients with CKD, not because of primary skin disease or systemic or psychological disease, or possible anticoagulant therapy that may cause pruritus [51]. Moreover, another frequent ADE/ADRs reported was that of nosebleed, which can also be attributed to the bleeding diathesis of ESRD patients [52].

The reported limitations of the current study were those of a relatively small study sample, but this can be compared against the aimed population—CKD patients of stage five—and is due to the location of the study in Chania prefecture, which has a population of approximately 180,000 people. However, it can be stated that the population of the study was representative of people with ESRD disease in Greece, as well as of those of other studies mentioned earlier. Another limitation was any information bias from the data gathered from patients. For this reason, diligence in informing the purpose and objectives of the study were employed, as well as the Naranjo scale, to further analyze the data. Finally, the aim of the study could be considered another limitation, since it is difficult to distinguish if the ADE/ADRs that are included in SOPs and national formularies of drugs are drug-related or disease-related in this case. This could be further clarified in future studies that compare ESRD cohorts with other patient cohorts with similar medication regimens. However, in the current approach, the aim was to record what type of ADE/ADRs can be recorded (either disease- or drug-related). In addition, in an effort to provide some data that are generally missing for Greece, another effort was made to analyze potential DDIs that could be related with ADE/ADRs in those patients due to the high number of drugs administered. These data could assist physicians, nurses, and pharmacists to improve the provision of healthcare in special population groups such as patients with ESRD.

Nowadays, the utilization of state-of-the-art technologies in clinical practice, along with the further understanding of biology and pharmacology, can assist medical personnel to improve their healthcare [53,54,55]. Aiming to reduce the risk of ADE/ADRs, the early detection of problems and good communication between healthcare providers and patients, regular review of treatment, continuing education, adoption of preventive measures, development of quality of care, organization in the work environment and the development of a critical view of uniform patient safety assessment standards are important parameters in modern therapeutic practice [22,56].

## 5. Conclusions

The current study tried to assess the impact of medication regimens on ESRD patients undergoing hemodialysis regarding ADE/ADRs in Greece. Overall, it was evident that the complexity of the medication administered, along with disease status, leads in the appearance of an increased number of side effects that can be attributed to increased drug action, DDIs or disease characteristics. It is evident that this special group of patients needs additional monitoring and advanced healthcare from all involved medical personnel to avoid any complications from the provided therapies and healthcare.

## Figures and Tables

**Table 1:** Strengthening the Reporting of Observational Studies in Epidemiology (STROBE) information for the study regarding methods and results.

Methods	
Study design	Analysis of DDIs ^1^ and ADE/ADRs ^2^ in ESRD ^3^ patients in Greece
Setting	Department of Nephrology of the General Hospital of Chania (GSC), in Crete Greece (6-month data collection).
Participants	Patients with ESRD that undergo hemodialysis 3 times per week and signed the informed consent form.
Variables	Record of demographic characteristics, comorbidities, medication regiments and laboratory test results; Analyze DDIs and ADE/ADRs as they are reported from the patients.
Data sources/measurement	DDIs and clinical significance based on a literature search of relative databases; ADE/ADRs: Greek National formulary of medicines, SOPs and European Medicine Agency (EMA); Naranjo algorithm.
Study size	Target population: All ESRD patients of Chania County in prefecture of Crete that visit GSC; Study population: Signed informed consent form to participate.
Bias	Diligence in informing the purpose and objectives of the study; Distinct questionnaires, patients were blind to each other; Record of demographics and medication (questionnaire No. 1); ADE/ADRs feedback (questionnaire No. 2); ADE/ADRs together without information if they are ESRD-related or specific-drug-related.
Results	
Participants	60 ESRD patients that signed informed consent form (95% of total ESRD patients in the clinic, with 100% follow-up for the two questionnaires).
Descriptive data	68.3% male and 31.7% female, mean age 64.9 years (51.7% elders > 65 years); Average years with ESRD: 4 years; Average medications: 10 per patient.
Outcome data	Comorbidities: Hypertension, Cardiovascular Disease and Diabetes; DDIs: 50 co-administered drug combinations (1–2 DDIs per patient), 8% of serious clinical significance; ADE/ADRs: average 16 ADE/ADRs. CNS-, GI-track- and Musculoskeletal-system-related ADE/ADRs were most often reported.
Main results	ADE/ADRs as clinical outcome from DDIs reported back in 64% of the total identified DDIs; 93% of reported ADE/ADRs are related with CNS, GI-track and musculoskeletal system; ADE/ADRs that are observed in ESRD can be also related to disease state in some cases.

^1^ DDIs: Drug-Drug Interactions; ^2^ ADE/ADRs: Adverse Drug Events/Adverse Drug Reactions; ^3^ End Stage Renal Disease.

**Table 2:** ADE/ADRs that are included in the study and included in Greek National Formulary and drugs’ SOPs.

Organ System	ADE/ADRs
Sensor organs (general)	change/loss of taste, tinnitus, blurred vision, dry mouth, eye pain, feeling of heat/cold (hands/feet), feeling of discomfort, hand numbness.
Respiratory	cough, bronchospasm, dyspnea, shortness of breath.
Gastro-Intestinal (GI) track	anorexia, diarrhea, vomiting, constipation, gastrointestinal bleeding, intestinal bleeding, weight loss, abdominal pain, flatulence, nausea, jaundice.
Immune	nasal drainage, flu feelings.
Integumentary system	rashes, itching, urticaria, hair loss.
Musculoskeletal system	weakness, restless legs, chest pain, painful muscle contractions, fatigue, myalgia, muscle weakness, arthralgia, tonic spasms, muscle cramps, trembling, weight loss.
Cardiovascular	arrhythmia, tachycardia, bradycardia, hypotension, hypertension.
Central Nervous System (CNS)	memory loss, insomnia, sleep disturbances, dizziness, vertigo, headache, lethargy, nervousness, drowsiness, hallucinations, nervousness, heat flushing, chills.

**Table 3:** Demographic characteristics and lab test results of ESRD patients in the study.

Demographics	Male (%)	Female (%)	Total (%)
Gender	41 (68.3%)	19 (21.7%)	60
Age (>65) (elder)	24 (77.4%)	7 (22.6%)	29 (48.3%)
Age (≤65) (adults)	17 (58.6%)	12 (41.4%)	31 (51.7%)
Physical characteristics	Height (meter ± SD) ^1^	Body weight/Dry weight (kg ± SD)	BMI ^2^ (kg/m^2^ ± SD) & BMI dry weight
	1.7 ± 0.1	88.4 ± 20.4/76 ± 17.8	30.6 ± 5.6 & 25.5 ± 3.4
Educational attainment	Primary	High School	University or higher
	28 (46.6%)	19 (31.6%)	13 (21.6%)
Socio-economic status	Low	Basic	Average-high
	6 (10%)	29 (48.3%)	25 (41.6%)
Residence area	Urban	Suburban	Rural
	26 (43.3%)	17 (28.3%)	17 (28.3)
Family status	Married	Single	Widowed/Divorced
	43 (71.6%)	10 (16.7%)	7 (11.6%)
Lifestyle	Smoking (%)	Alcohol	Coffee
	22 (36.7%)	5 (8.3%)	37 (61.7%)
**Lab test results (mean ± SD)**			
Urea mg/dL	128.9 ± 28.1 (before dialysis); 34.7 ± 13.4 (after dialysis)		
Creatinine mg/dL	9.1 ± 1.9 (before dialysis); 3.4 ± 1.0 (after dialysis)		
KCl mEq/L	5.3 ± 0.7 (before dialysis); 3.6 ± 0.7 (after dialysis)		
SGOT IU/L	14.1 ± 4.7		
SGPT IU/L	14.3 ± 6.0		
γGT IU/L	24.0 ± 28.3		
Hemoglobin g/dL	11.6 ± 1.8		
Hematocrit %	36.4 ± 4.2		
CaCl mg/dL	9.1 ± 1.0		
Phosphate ng/mL	5.3 ± 1.7		
Ferritin ng/mL	462.9 ± 242.2		

^1^ SD: Standard Deviation; ^2^ BMI: Body Mass Index.

**Table 4:** Drug–drug interactions detected among medication regimens of ESRD patients, their clinical significance, and cases of reports of relative ADE/ADR.

Combination	Drug A	Drug B	Type	Mechanism	Drug Affected	Result	Significance	Report Relative ADE/ADR (No. of Patients)
1	sucroferric oxyhydroxide	levothyroxine	PK ^1^	GI ^3^ absorption	levothyroxine	reduced F ^4^	Serious	YES (7/11)
2	amiodarone	carvedilol	PD ^2^	Synergism	both	bradycardia	monitor	YES (2/2)
3	amiodarone	acenocoumarol	PD	synergism	acenocoumarol	INR ^5^ increase	moderate	YES (1/1)
4	nifedipine	atorvastatin	PK	CYP ^6^ 3A4 & Pgp ^7^	atorvastatin	statin-ADRs ^8^	monitor	YES (1/1)
5	azithromycin	atorvastatin	PK	CYP3A4	atorvastatin	statin-ADRs	monitor	YES (1/1)
6	nifedipine	alprazolam	PK	CYP3A4	alprazolam	increased action	moderate	YES (1/1)
7	aspirin	celiprolol	PD	Synergism	both	Increase K+	moderate	NO (0/1)
8	aspirin	bemiparin	PD	synergism	both	bleeding	moderate	NO (0/1)
9	omeprazole	clopidorgrel	PK	CYP2C19	clopidogrel	ADRs	moderate	YES (2/10)
10	metronidazole	atorvastatin	PK	CYP3A4	atorvastatin	statin-ADRs	monitor	YES (1/1)
11	aspirin	sertraline	PD	platelet-serotonin	both	bleeding	monitor	NO (0/1)
12	aspirin	insulin	PD	insulin sensitivity	insulin	hypoglycemia	moderate	NO (0/2)
13	aspirin	carvedilol	PD	prostaglandin synthesis	carvedilol	reduced action	moderate	NO (0/6)
14	methylprednisolone	levofloxacin	PD	unknown	levofloxacin	tendinitis	monitor	YES (1/1)
15	methylprednisolone	monofloxacin	PD	unknown	monofloxacin	tendinitis	monitor	YES (0/1)
16	aspirin	clopidogrel	PD	synergism	both	bleeding	monitor	YES (5/12)
17	omeprazole	alprazolam	PK	CYP2C19	alprazolam	PD enhance	moderate	YES (1/1)
18	fluoxetine	clopidogrel	PK	CYP2C19	clopidogrel	PD reduction	serious	YES (1/1)
19	fluoxetine	aspirin	PD	platelet-serotonin	aspirin	PD enhance	monitor	NO (0/1)
20	fluoxetine	pregabalin	PD	reduced seizure threshold	pregabalin	PD reduction	moderate	NO (0/1)
21	gabapentin	alprazolam	PD	Synergism	both	PD enhance	monitor	YES (1/1)
22	alprazolam	codeine	PD	Synergism	both	sedation	moderate	YES (1/1)
23	sevelamer	ciprofloxacin	PK	GI absorption	ciprofloxacin	PD reduction	moderate	NO (1/1)
24	omeprazole	ciprofloxacin	PK	GI absorption	ciprofloxacin	PD reduction	moderate	NO (1/1)
25	aspirin	ciprofloxacin	PD	GABA ^9^	ciprofloxacin	CNS ^10^ ADRs	moderate	YES (1/3)
26	ticlopidine	codeine	PK	CYP2D6	codeine	PD reduction	moderate	YES (1/1)
27	prednisolone	aspirin	PD	increase anti-coagulation	aspirin	PD reduction	moderate	NO (0/2)
28	aspirin	enoxaparin	PD	Synergism	both	bleeding	moderate	YES (1/3)
29	alprazolam	pregabalin	PD	Synergism	both	ADRs	monitor	YES (1/1)
30	prednisolone	fondaparinux	PD	increase anti-coagulation	fondaparinux	bleeding	monitor	NO (0/1)
31	metoprolol	carvedilol	PD	Synergism	both	PD enhance	Serious	YES (1/1)
32	aspirin	lornoxicam	PD	Synergism	both	PD enhance	monitor	YES (1/1)
33	gabapentin	codeine	PD	Synergism	both	PD enhance	monitor	NO (1/1)
34	levothyroxine	acenocoumarol	PD	Synergism	acenocoumarol	PD enhance	monitor	YES (1/1)
35	nadroparin	acenocoumarol	PD	Synergism	both	PD enhance	monitor	YES (1/1)
36	sevelamer	ciprofloxacin	PK	GI absorption	ciprofloxacin	PD reduction	serious	NO (0/3)
37	calcium carbonate	ciprofloxacin	PK	GI absorption	ciprofloxacin	PD reduction	monitor	NO (0/2)
38	furosemide	amikacin	PD	Synergism	both	PD enhance	moderate	YES (1/1)
39	calcium carbonate	levothyroxine	PK	GI absorption	levothyroxine	PD reduction	monitor	NO (0/2)
40	omeprazole	ciprofloxacin	PK	GI absorption	ciprofloxacin	PD reduction	moderate	NO (0/2)
41	insulin	ciprofloxacin	PD	glucose homeostasis	insulin	PD-ADRs	monitor	YES (1/2)
42	cinacalcet	codeine	PK	CYP2D6	codeine	PD reduction	moderate	YES (1/2)
43	ciprofloxacin	vildagliptin	PD	Synergism	vildagliptin	PD enhance	monitor	YES (1/1)
44	tramadol	pregabalin	PD	Synergism	both	PD enhance	monitor	YES (1/1)
45	azithromycin	nardoparin	PD	increase anti-coagulation	nardoparin	PD enhance	moderate	NO (0/1)
46	azithromycin	sunitinib	PD	QT-prolongation	sunitinib	PD enhance	monitor	YES (1/1)
47	fenofibrate	insulin	PD	insulin sensitivity	insulin	hypoglycemia	monitor	NO (0/1)
48	omeprazole	escitalopram	PK	CYP2C19	escitalopram	PD-adrs	monitor	YES (1/1)
49	lornoxicam	escitalopram	PD	Synergism	both	bleeding	moderate	NO (0/1)
49	clopidogrel	escitalopram	PD	platelet activation	clopidogrel	bleeding	monitor	YES (1/1)
50	gabapentin	escitalopram	PD	seizure threshold	gabapentin	sedation	monitor	YES (1/1)

^1^ PK: Pharmacokinetic; ^2^ PD: Pharmacodynamic; ^3^ GI: gastro-intestinal; ^4^ F: Bioavailability; ^5^ INR: International Normalized Ratio; ^6^ CYP: Cytochrome P450; ^7^ P-gp: P-glycoprotein; ^8^ ADRs: Adverse Drug Reactions; ^9^ GABA: Gamma-Amino Butyric Acid; ^10^ CNS: Central Nervous System.
